# Notes of an European Tour

**Published:** 1845-12-01

**Authors:** F. H. Hamilton

**Affiliations:** Professor of Surgery in Geneva Medical College


### BUFFALO MEDICAL JOURNAL

**Vol. 1.** DECEMBER 1, 1845. **No. 7.**

### Notes of an European Tour.---No. 7.

Communicated for the Buffalo Medical Journal, by F. H. HAMILTON, M. D., Professor of Surgery in Geneva Medical College.

### Italy.

It was past three, when the servant knocked to say that the coach was nearly ready; and remembering that to-day, I was to cross the Alps I sprang upon my feet with all the buoyancy and exhilaration of a boy who wakes to a long anticipated gala-day. The Simplon pass is among the highest of those which traverse the Alps; but as it affords a very direct communication between this part of Switzerland and the north of Italy, it has long been used as a "courier track," though often at the peril of life, being always subject to floods and avalanches, and having been nearly blocked up with fragments of rocks by the earthquake of 1755, which destroyed Lisbon.

The present road, one of the greatest architectural achievements in the world, is, in fact, but a link of the same great road which extends from the walls of Paris, across the Jura mountains, through Geneva, up the valley of the Rhone, and which is terminated at the triumphal gate of Milan; most of which was built under the direction of Napoleon, to enable him the better to accomplish his purpose—the subjugation of Italy. But of all this chain of splendid road, this link is the *chef d'œuvre*, in the construction of which 3000 men were employed nearly six years. Its length from Brieg to Domo d'Ossola is forty miles; its breadth twenty-five to thirty feet: and in addition to the almost continuous line of masonry, which as a foundation and parapet wall protects its lower side, and the numberless subterranean sluiceways, &c., there are ten galleries or tunnels, hewn mostly from the hardest granite; more than fifty bridges, twenty stone buildings stationed at intervals as "post houses," dwellings for the "cantonniers" who keep the road in repair, and places of "retuge" for such as are overtaken by storms.

It was four o'clock when we left Brieg, and the gray twilight was just beginning to appear between the summits of St. Gothard and Finster-Aar-Horn, faintly opening to view the valley of the Rhone as it mounts up to its source in the glaciers of Gallenstock and the gloomy. "Lake of the Dead."

Our course, after having made a slight detour around the base of a small hill crowned with several neat chapels and oratories, lay chiefly along the right bank of the Saltine, whose bed, swollen by the warm sun of the two preceding days, we could hear dashing through the deep gorge below us. At the third "refuge" we stopped to breakfast, and strolling out while coffee was preparing, I was attracted by the voice of a little Swiss girl about eight years old, who held in a basket a few Alpine minerals, which she was offering for sale. I could not understand a word she said, yet there was language in her weary step, in her rags, and in her joyless face: it was the same language of want, and penury, and disease, to which my eyes had already become familiar in the Vallais canton.—She was the daughter of a Shepherd, I was told, who lived about two miles distant, and at this early hour she had come up the mountain to meet the "post." I selected from her little cabinet such as were most rare, and having paid her all she demanded, we parted mutually pleased with the exchange.

Breakfast over, I threw my overcoat into the coach and hurried off, determined to walk up the remainder of the mountain. Passing through two or three long galleries, I entered at length, beyond the fifth refuge, the first of the "glacier" galleries, constructed so as to allow the avalanche, as well as the water which at this season pours in torrents from the glaciers above, to pass over and precipitate themselves into the chasm below. The constant dripping through the fissures above has lined the roof and sides with large and fantastic icicles, which the greatest heat of midsummer fails to loosen. The air was cold and chilling in the extreme, and I hastened to escape. Emerging from the last gallery, I was glad to feel again the influence of the sun, which was now fairly in the heavens, and unobscured by a single cloud, began even at this elevation to impart a sensible and genial warmth: and especially did it seem the more welcome amid the scene of utter desolation which now presented itself. On each side of the road, the snow lay piled in masses, extending on the left far up into the perpetual glaciers of Mont Leone, while deep below, on the right, the Saltine was seen foaming and struggling to force the barriers formed by the rocks and uprooted trees which the recent "snow-slide" had borne down in its course. Behind, overlooking the valley of the Rhone, the snowy tops of the Bernese Alps lay in full view, the extensive glacier of Aletsch reflecting the sun with dazzling brilliancy from its summit almost to its base. There is an awful sublimity in such desolation, where, on all sides, nothing is seen but mountains covered with an everlasting drapery of ice, or rocks whose hoary heads are blasted and shivered into irregular turrets, and upon whose slippery sides scarcely the tuft of moss can find a footing. Where nothing is heard but the roar of the mountain-torrent, or the distant growling of a bursting glacier, or the rush of the avalanche—sounds which, on a spring morning when the sun is warm and clear, constantly fall upon the ear like successive peals of heavy but distant thunder.

I stood riveted by the novelty and grandeur of the scene, until the "post" had nearly overtaken me, when, remembering that I had not yet accomplished my vow, I turned and hurried on until I had passed the last refuge on this side of the mountains. The road had now become slippery and the ascent more difficult: and, sheltered from the winds, I had thrown open my coat and vest, and loosened my cravat, and was beginning to complain of the uncomfortable heat of an Alpine climate, and to doubt how I might endure the greater heat of the southern slope, when, as I turned up through the defile which opens to the opposite side of the mountain, I was met by a snow-storm which came pelting upon me in a most unmerciful manner. The wind was blowing almost directly from the south: it was the same fiery simoon which had scorched the traveller upon the sands of Egypt, and had warmed the limbs of the basking lazzaroni upon the shores of Italy, and licked up the moisture from its sodden soil, that now gave me so sharp a reception; but on its way it had kissed the icy brow ef Mont Rosa and Cervin, and its breath had become cold and piercing as a winter's blast. The spot on which I stood extended on all sides a bleak, deserted waste, and the road could only be recognized by the poles set in the ground, at short distances, to serve as guides. A wooden cross, bearing a rude inscription, announced that I had reached the highest point of the pass: I had accomplished my vow, and would now gladly have retreated to the shelter of the carriage: but it was too far behind to allow me to wait either for it or my overcoat. I had no choice but the convent about half a mile ahead: so, buttoning close and leaning forward, I commenced a regular tilt against the storm, and despite the disadvantage of being obliged to run with my eyes shut, and an occasional plunge into a snow bank, to which my blind, zig-zag course subjected me, I soon measured the distance.

As I came up to the hospice, four or five large St. Bernard dogs sprung playfully towards me, as if to welcome me to their charitable shelter.—I gave the noble animals my hand, and passing, ascended the long flight of stone steps which leads to the main entrance. The present building is a large stone edifice, larger, I am told, than the St. Bernard hospice, and but recently finished: but the old convent, a ruined tower of which is seen in the valley below, was built many centuries since, when there was nothing but a mule track across the mountains. The new building contains a chapel, drawing-room, dining-room, kitchens, &c., with a large number of sleeping apartments, Those which I entered were large and richly furnished. The beds were luxurious in the extreme.

The monks, three or four in number, are, like those upon the great St. Bernard pass, of the order of St. Augustine. Father Barras, the present excellent prior of the Simplon convent, was for more than thirty years an inmate of the St. Bernard. It is the duty of these brethren to receive and entertain all such as have no means to pay for their accommodations, or are unable to proceed from severity of weather or other cause. After a storm, one or more, accompanied with a servant and their faithful dogs, go out over all the usual mountain paths, where travellers or the bold chamois hunters are known to cross, or where "slides" are accustomed to occur, or wherever the country is peculiarly exposed to hurricanes: and not a winter has passed since the recollection of their oldest brother, but their self-denying toil has been rewarded by some person thus rescued from a fearful death.

From the convent a rapid drive of three miles brought us to the small village of Simplon, where we dined most sumptuously upon fresh eggs and mountain trout. After dinner, and while the horses were being harnessed. I walked over to the chapel which stands nearly opposite the post-house, and looking through an open door at the side, I saw a small room hung round with pictures of martyrs, the virgin Mary, &c., at each end of which was a pile of human bones, laid up carefully and regularly to the height of five feet against the walls. I am an amateur in such things, and I enjoyed the sight as an artist would a genuine Correggio or Titian. Clean, white and marrowless, skulls curiously shaped, with short and crooked tibia's—what a prize for an anatomical cabinet! Why these bones were piled here, I could never learn, but only that they had all been once buried decently and comfortably, and at some time were taken up and placed here to shiver and chatter their teeth in the cold.

When I returned the coach was waiting, and fastening the wooden sabot under the wheels, we continued our descent amidst a cold, dense cloud of fog—so variable is the air near the summit of the mountain. Driving some distance along the right bank of the Vedro, whose channel we were now following, by a sudden turn we entered the gallery of Algaby, beyond which we crossed by a lofty bridge to the opposite side of the gorge. The mountain now began to crowd upon us, and the pass became more and more narrow at every foot—becoming also deeper and darker and more fearful, as we descended. The road is here chisseled along the sides of the cleft, or bored through the abrupt angles with almost incredible labor and skill; in all its windings and crossings smooth and solid and secure; yet no one has ever traversed, for the first time, this celebrated " gorge of Gondo," said to be one of the most grand and savage in the Alps, without emotions of awe amounting almost to fear.

In a deep and gloomy chasm directly below, rushes the torrent of the Vedro: above, on either side, arise two vertical walls, black and dripping with moisture, and from many points of which the half-loosened masses seem just ready to fall. Thus hemmed in by colossal walls, which scarce admitted the light of day, stunned by the noise of the dashing of the waters below, and blinded by the whirling clouds of spray, it seemed the very entrance to the infernal regions—an impression which was not at all diminished when we drove at full speed into the dark mouth of the cavern which forms one of the most remarkable features of this pass.

Beyond this gallery, the grandeur of the scenery, unaltered, except by the still greater depth and height, and still lessened breadth, suffers no abatement. Near by is the hamlet of Gondo, a pile of rough stone cabins in the very depth of the pass, grouped around the base of a singular and lofty building which I fancied might have been constructed as a guard-house. Crossing the frontier we were stopped at the Sardinian custom-house, to show our passports and submit to an examination of luggage. The examination was a mere form, and after a few minutes' detention, we were permitted to drive on.

We were now at the end of the gorge, and as the mountains gradually receded, the mists cleared away and the air became less chilling; the dark foliage of the Alpine pines gave place to the rich and the more delicate tints of the beech and chestnut. Green patches of grass were also seen scattered here and there upon the warm southern slopes. But it was not until the valley of the Toccia burst in view, into whose bed the Vedro at length empties itself, that I felt fully the glory and sweetness of an Italian scene. Oh! it is beautiful beyond description, to one who has come from the mouldering towns and sterile soil of the Vallais, and crossed the stormy summits of the Alps, and passed the horrible gorge of Gondo, to look thus suddenly upon the sunny plains of Italy. The change is like enchantment. The sun is more bright, the air is softer, and the sky assumes a warmer hue. A rich lawn of velvet spreads up towards the mountains, and neat white villages are scattered here and there along the valley, or beautifully terraced upon the hill-sides. Elegant villas, with white stuccoed walls, and green verandahs, and light balconies, stand amidst groves of olives and fields of trellaced vines.

It was amidst such scenes and under such circumstances, that I entered Italy for the first time: and it seemed a picture of unsurpassing beauty and loveliness—a very paradise! To-day I have seen *more* of Italy.—The picture has a reverse, with strange and painful contrasts. We slept at Domo d'Ossola.

I was out at early dawn and rambled long through this ancient town of the Lepontii, before the beggars began to gather; it is the only hour when the traveller can enjoy, unmolested by human misery, an Italian sky.—And as one after another they came up from their cellars, or from under the sheds and holes where they had slept, I was surprised, and almost annoyed to recognize upon many of them my late digusting acquaintance, the goitre. I hoped I had left it behind me forever, beyond the Alps, but it was here haunting me again, yet much less universal, and without the dreadful accompaniments of Switzerland. A Pharmacien upon whom I called, told me that with a population of nearly 3000, Domo d'Ossola had not more than four cretins.

Late in the morning I saw an old man laying in the sun upon a stone seat near a public fountain; he seemed asleep, and I thought it would be well if he slept and was buried, for he was a sight so loathsome—he was a "pellagrose."

From Domo d'Ossola to Milan is a single day's journey, and the road all the way is excellent. At Milan I made my first visit to the celebrated "Ospedale Maggiore," one of the most extensive hospitals in Europe, built by the liberality of a Noble, in 1456. The entire building forms a square, of which the front facade, 800 feet in length, stands directly on the street. To Dr. Antoine Capelli, one of the physicians to this princely establishment and a member of the "Washington society," I am indebted for many attentions during my short stay at Milan, and especially for much information in relation to the pellagra, of which more cases are always found in this charity than any where else in Italy. It is at this season of the year especially that the hospital becomes crowded, the disease being almost uniformly aggravated in the spring. Dr. Capelli kindly showed me the malady in all its phases and degrees of severity, explaining to me its usual progress. It attacks chiefly the peasantry, especially females, and not usually until after middle life. In the earliest stage the patient complains only of a general mauvaise, accompanied with some headache, pain at pit of stomach and occasional diarrhœa. This continues frequently during all the spring and summer, but disappears in autumn. On the following spring the same train of symptoms is renewed, but with increased violence, and now a shining eruption appears in patches upon the back of the hands, face, &c., which is soon covered by minute squamæ, the skin becoming thick nnd rough. Hereafter during the winter the eruption generally improves, and also the disturbance of the digestive organs, yet from year to year the eruption is extending and the disease becoming regularly more confirmed and complicated: the limbs are bloated, the abdomen distended, spasms and convulsions occur, followed by delirium, but more generally complete coma closes the scene. From the very inception a peculiar mental depression, often amounting to absolute despair, is present, and which I was able to note in its various degrees in my walks through the wards: some looked sad, others were morose and would not speak, one woman about 30 years of age, who had for some time refused medicine, to-day declared that she would not again take food, fully determined to put an end to an existence which, she was convinced, must always be miserable. "She has indeed," said Dr. Capelli, "too much reason to think so, for it is very rarely when the disease has lasted two or three years that the patient ever recovers, he may perhaps live to an old age, but he is always a loathsome pellagrose." It was cheering, however, among all this multitude of leprous patients to see one cheerful face. A girl, about 18 years old, much younger than the period at which it usually commences, was pronounced convalescent and would in a few days be dismissed cured; and her face was lighted with a smile of recognition and gratitude as the Doctor passed.

The causes of this scourge of Lombardy are, I am convinced, and so also was I assured by Dr. C., to be found not solely in the low, damp soil of the adjacent country, and the exposure to a burning sun, but chiefly in the peculiarly unwholesome diet of the peasantry, whose food, exclusively vegetable, is chiefly composed of indian flour made into a coarse and always acid bread, such as I have frequently eaten in Lombardy, and which never failed to put my stomach into a fermentation; or indian flour and chestnut flour stirred into a porridge called "polenta," and to which the rye porridge of Scotland is not unlike. Their wine also, such as I was able to get, was very sour. In addition to this, they are weighed to the ground by a despotism, civil and religious, no where else surpassed, and which sinks both mind and body into the lowest degradation; and however much government or private liberality may do to establish "maisons de sante," public baths, or to prevent intermarriage among pillagrous persons, or even to drain by canals and trenches, the vast plains of Lombardy, which Napoleon boldly projected and even commenced, still until some effectual means to elevate the condition of the peasantry are taken, little will be gained. The history of a Lombard peasant is,—born in a hovel, reared in a shed, oppressed by the government and robbed by the priest; through all his life laboring like a beast and not half as well fed, and dying at last a miserable pellagrose! For all this Austria has to answer, and must answer sooner or later.

The treatment of the pellagra at this hospital consists mainly in a plain, healthy, nutritious diet and *baths*. These latter are considered indispensable.

